# Concordance on zebra stripes: a comment on Larison *et al.* (2015)

**DOI:** 10.1098/rsos.150323

**Published:** 2015-09-30

**Authors:** Tim Caro, Theodore Stankowich

**Affiliations:** 1 Department of Wildlife, Fish and Conservation Biology, University of California, Davis, CA 95616, USA; 2 Department of Biological Sciences, California State University, Long Beach, CA 90840, USA

The functional significance of the extraordinary black and white stripes of zebras is still mysterious but now an active field of research. Four major hypotheses have been put forward: stripes are an antipredator defence operating through crypsis [1] or confusion of predators [2], are a means of reinforcing social bonds [3], are defence against ectoparasites [4] or are a means of cooling zebras [5]. Now, in the second multifactorial analysis of striping in zebras to date, Larison *et al.* [6] investigated the environmental factors that explain geographical variation in striping within a single species, the plains zebra (*Equus quagga* or *Equus burchellii*). They matched variation in striping patterns at 16 sites across its geographical range to a suite of environmental variables as well as tsetse fly (glossinid) distribution and lion (*Panthera leo*) presence. They found that greater intensities of intraspecific striping were associated with warmer temperatures and high precipitation.

Last year we published a similar but phylogenetically controlled analysis using all seven species of equids, namely the three striped zebra species, the African wild ass (*Equus africanus*) which has leg stripes but no body stripes, and the unstriped species of equids (*E. hemionus, E. kiang, E. ferus przewalski*), and 20 of their subspecies, and compared several aspects of the four hypotheses [7]. These were camouflage in woodlands, antipredator defence against both lions and spotted hyaenas (*Crocuta crocuta*), social interactions, tsetse fly distribution, tabanid distribution using both temperature and humidity ranges as a proxy for abundance, and temperature isoclines to assess the cooling hypothesis. First we ran univariate tests on individual factors to identify the hypotheses with the strongest predictive ability, then subsequently used AICc model selection procedures to pit the most promising predictors against each other—a powerful statistical protocol for testing multiple hypotheses. Further, our study was corrected for phylogenetic relatedness between the species and subspecies, allowing us to draw conclusions about correlated evolution between body striping and environmental selective pressures. Our study provided strong evidence that our proxy for tabanid fly parasitism was essentially perfectly correlated with the presence of stripes across species across almost every area of the body, and especially for leg stripes, the level at which biting flies prefer to land on hosts.

Our comparative study pertains to the factors that maintain striping across species of equids, whereas Larison and co-workers' conclusions are specific to the ecocorrelates of variation in striping within plains zebras, and cannot necessarily be extrapolated directly to infer the selective pressures that favoured the original evolution of zebra stripes, which only occurred once in the genus. That said, these two studies, conducted independently of each other, have now tested a number of hypotheses for the function of striping in zebras, with both studies using a multifactorial approach that pits the hypotheses against each other allowing for direct tests of multiple hypotheses within the same model. Here we want to stress that these two studies agree on a number of points that move detective work on the mystery of zebra stripes forward considerably.

With regard to individual hypotheses, both studies found no convincing evidence for the geographical distribution of stripes on any part of the body being associated with lion distribution across the African continent. Both studies remark that this is unsurprising because a considerable body of literature finds that zebras are a preferred prey of lions in most ecosystems [8]. Taken together, our findings therefore cast serious doubt on whether experiments involving human subjects capturing striped objects moving across computer screens (analogies for lions chasing zebras) are worthwhile tests of the adaptive significance of striping in zebras. Second, our studies found no evidence for striping being associated with woodland or tree cover, refuting the idea that stripes are a form of crypsis against predators. Third, neither study found strong evidence for variation in striping being related to glossinid (tsetse) distributions. The only significant association was between belly stripe number and tsetse flies in Caro *et al*. [7]; none was found for other parts of the body in either analysis.

The studies additionally concur in finding aspects of striping being related to temperature and moisture. We found many aspects of striping (number of face stripes, neck stripes, flank striping, rump striping, shadow stripe severity and leg stripe intensity) are associated with temperatures lying between 15°C and 30°C and humidity between 30 and 85% (a proxy for tabanid abundance) when these conditions persist for a minimum of six to seven months. Note, such warm humid conditions are congruent with Larison *et al.'s* precipitation during the wettest month of the year (BIO13 in WorldClim) and constant annual temperatures (BIO3) which they found were associated with striping measures in plains zebras. In short, the studies find that striping is associated with warm humid conditions both inter- and intraspecifically.

Where the studies differ is in their interpretation of these results. Larison and co-workers focus on their temperature rather than on their rainfall association and discuss how temperature is correlated with striping measures in plains zebras although they are agnostic as to mechanism. While acknowledging that stripes might set up convection currents over the torso [5] and so cool the animal, in regards to the fore and hindlegs where they found positive associations with temperature too, they write ‘The association between temperature and striping on the legs is not easily explained as a mechanism for thermoregulation. It may simply be a result of genetic correlation as stripe characteristics on the legs and torso are highly correlated, or it may be a response to a different mechanism, such as avoiding biting flies’. We would suggest that as leg stripes but not body stripes are found in non-zebra species such as *E. africanus* that are under heavy tabanid pressure, body stripes might stem from genetic correlation with leg stripes since more species have leg stripes than body stripes.

Caro and others suggest that striping is associated with tabanid and possibly other biting fly annoyance. There is experimental evidence that tabanids eschew landing on white [9], striped (e.g. [4,10]) and spotted surfaces [11], that zebra stripe widths are thinner than those on which horse flies, stable flies and tsetse flies prefer to land, that zebra pelage is thinner than the length of biting fly mouthparts making them susceptible to blood loss, and because several groups of biting flies carry diseases fatal to zebras [7]. By contrast, there is no experimental evidence as yet for or against the setting up of convection currents by stripe patterns over the zebra dorsum or other body areas. Moreover, against cooling being the primary selective pressure driving black- and white-striped pelage, we would add that thick saturated black stripes that absorb radiation more than thinner, less saturated black stripes or white stripes would be unlikely to be found in hotter climates if there is a premium on staying cool; that any shadow stripes lying between deep black stripes would disrupt reflective properties of white stripes; and that convection currents would be ineffective on windy floodplains often inhabited by plains zebras or when animals are moving. Indeed, like artiodactyls [12], we would expect other ungulates to wear entirely light-coloured coats of high albedo in hotter environments. Finally, measurements of free-living plains zebras and sympatric ungulates using an infrared camera show that zebras are always significantly warmer than three sympatric herbivores photographed under the same conditions: mean flank temperatures, zebra 36.0°C (*N*=57 individuals), impalas *Aepyceros melampus* 34.2°C (*N*=49), buffalo *Syncerus caffer* 34.1°C (*N*=35) and giraffe *Giraffa camelopardalis* 33.3°C (*N*=27) [13].

Interestingly, Larison and her co-workers suggest that trypanosomes and other diseases carried by tsetse flies may have a compromised ability to develop at colder temperatures, reminding readers that the distribution of biting flies may have less relevance than the distribution of diseases carried by those flies. They concede that their temperature-striping associations could reflect favourable conditions for infectious diseases carried by biting flies that adversely affect zebras: ‘We suggest that temperature may influence trypanosome prevalence in tsetse flies and as a consequence help explain variation in striping’.

Caro and others tested an additional hypothesis interspecifically that the Larison group did not test intraspecifically. This was striping being associated with mean and maximum group sizes as a proxy for social interactions, for which we found no evidence.

While it is often expedient to call attention to differences in findings between studies, in the interests of publication and publicity, it is often more constructive in the long run to focus on similarities. Here, we suggest that the only two multifactorial studies of this problem (table 1), conducted independently, have remarkable concordance in (i) dismissing lion predation as driving striping in equids, (ii) rejecting stripes having a cryptic function in wooded habitats, (iii) casting aspersions on tsetse fly distributions being of major importance for striping, and (iv) either questioning [6] or dismissing [7] the idea that stripes can generate cooling eddies, respectively, on the legs or anywhere on the body, and (v) indirectly point to tabanid or other biting fly (e.g. muscid, simuliid or mosquito) annoyance being the evolutionary driver of striping in Equidae, most probably because of the diseases that they carry and for which they are renowned [13].

**Table 1.** Summary of findings from the two multifactorial studies of striping in zebras.

hypothesis	comparative test: Caro *et al*. [7]	intraspecific test: Larison *et al*. [6]	notes
*predation*			
crypsis	no	no	no association with trees
spotted hyaena	unlikely	not tested	
lion	no	no	zebras are preferred prey
*social interaction*	no	not tested	all equids highly social
*antiparasite*			
glossinid	no (belly yes)	no	carry diseases fatal to equids and do not like to land on stripes
tabanid	yes	not tested	carry diseases fatal to equids and do not like to land on stripes
*cooling*	no	yes	mechanism unclear: includes both temperature regulation and disease

The studies' joint handicap, as both sets of authors explicitly recognize, is that we are hampered by lack of understanding of the extent to which temperature and humidity predict fly annoyance, the specific problems posed by biting flies, the extent to which biting fly distributions and the diseases that they can carry elide, and the mechanism by which stripes prevent flies from landing on zebra pelage mediated through disruption of outline, modulation of brightness or of polarized light [4], or confusion of insect motion detection systems controlling their approach and landing [14]. We suggest that future research targeted at these issues will improve our understanding of why zebras have black and white stripes.
